# Facing hierarchy: a qualitative study of residents’ experiences in an obstetrical simulation scenario

**DOI:** 10.1186/s41077-022-00232-1

**Published:** 2022-10-23

**Authors:** Adam B. Garber, Glenn Posner, Taylor Roebotham, M. Dylan Bould, Taryn Taylor

**Affiliations:** 1 Department of Obstetrics and Gynecology, The Ottawa Hospital Civic Campus, University of Ottawa and The Ottawa Hospital, 1053 Carling Avenue, 4th floor, D4, Ottawa, Canada; 2 Department of Obstetrics and Gynecology, Department of Innovation in Medical Education, The Ottawa Hospital Civic Campus, University of Ottawa and The Ottawa Hospital, Loeb Research Building — 1st floor, 725 Parkdale Ave, Ottawa, ON K1Y 4E9 Canada; 3 Department of Obstetrics and Gynecology, London Health Sciences Centre-Victoria Hospital, Western University, B2-401, London, ON N6H 5W9 Canada; 4 Department of Anesthesiology and Pain Medicine, Department of Innovation in Medical Education, University of Ottawa and the Children’s Hospital of Eastern Ontario, 401 Smyth Rd, Ottawa, ON K1H 8L1 Canada; 5 Department of Obstetrics & Gynecology, Centre for Education Research & Innovation, Schulich School of Medicine & Dentistry, London Health Sciences Centre-Victoria Hospital, B2-401, London, ON N6H 5W9 Canada

**Keywords:** Simulation, Power, Hierarchy, Residents, Surgery, Ob/Gyn

## Abstract

**Background:**

Residents in surgical specialties face a steep hierarchy when managing medical crises. Hierarchy can negatively impact patient safety when team members are reluctant to speak up. Yet, simulation has scarcely been previously utilized to qualitatively explore the way residents in surgical specialities navigate this challenge. The study aimed to explore the experiences of residents in one surgical specialty, obstetrics and gynecology (Ob/Gyn), when challenging hierarchy, with the goal of informing future interventions to optimize resident learning and patient safety.

**Methods:**

Eight 3rd- and 4th-year Ob/Gyn residents participated in a simulation scenario in which their supervising physician made an erroneous medical decision that jeopardized the wellbeing of the labouring mother and her foetus. Residents participated in 30–45 min semi-structured interviews that explored their approach to managing this scenario. Transcribed interviews were analysed using qualitative thematic inquiry by three research team members, finalizing the identified themes once consensus was reached.

**Results:**

Study results show that the simulated scenario did create an experience of hierarchy that challenged residents. In response, residents adopted three distinct communication strategies while confronting hierarchy: (1) messaging — a mere reporting of existing clinical information; (2) interpretive — a deliberate construction of clinical facts aimed at swaying supervising physician’s clinical decision; and (3) advocative — a readiness to confront the staff physician’s clinical decision. Furthermore, residents utilized coping mechanisms to mitigate challenges related to confronting hierarchy, namely deflecting responsibility, diminishing urgency, and drafting allies. Both these communication strategies and coping mechanisms shaped their practice when challenging hierarchy to preserve patient safety.

**Conclusions:**

Understanding the complex processes in which residents engage when confronting hierarchy can serve to inform the development and study of curricular innovations. Informed by these processes, we must move beyond solely teaching residents to speak up and consider a broader curriculum that targets not only residents but also faculty physicians and the learning environment within the organization.

**Supplementary Information:**

The online version contains supplementary material available at 10.1186/s41077-022-00232-1.

## Background

Hierarchy, the presence of an authority gradient across members of the healthcare team, is longstanding and deeply embedded in both healthcare provision and training [1, 2]. Surgical residents perceive a steep hierarchy and often fail to speak across it [3]. Bullying of trainees appears to be disproportionately more prevalent in surgical specialties, and the degree of hierarchy, high clinical risk, high acuity, and the litigious nature of the field all contribute to reinforcing an unforgiving culture during training [4]. In addition to its negative impact on learning, this climate deters trainees from raising patient safety concerns in the most pressing moments of clinical care, when their contribution to the healthcare team can be most valuable [5].

Simulation has been utilized as a means to explore social and cultural phenomena within healthcare teams. Calhoun et al. found that high-fidelity or theatre-based simulation can reapproximate the complex social phenomenon of clinical hierarchy in an educational setting resulting in similar decision-making errors [6]. A quantitative study using simulation to evaluate anaesthesia residents’ ability to challenge an erroneous supervising physician found that residents struggled to advocate in both steep hierarchical and flat hierarchical environments [7]. In the follow-up qualitative study, residents described the negative impact of hierarchy on their wellbeing, their learning, and the safety of their patients. The authors also identified coping mechanisms that residents employed when dealing with hierarchy, including conflict avoidance and diffusion of responsibility [8]. In contrast, simulation has scarcely been utilized as an exploration and teaching means regarding hierarchy faced by surgical residents.

Obstetrics and gynecology (Ob/Gyn) residents are frontline care providers. They must frequently respond to medical crises in the context of a complex multidisciplinary healthcare team. There is not, at this point, an in-depth understanding of how perceptions of hierarchy affect Ob/Gyn residents’ performance in the team or of the mental processes and strategies they employ when faced with speaking across a hierarchy for the sake of optimizing patient care.

Speaking up during a medical crisis can be regarded as an ethical imperative on account of its proven impact on patient outcomes [9, 10]. Yet, the optimal approach to address this with respect to surgical trainees in the context of the embedded hierarchy remains uncertain. The present study aimed to contribute to this effort by using a qualitative design to explore, first, the utility of a hierarchy-focused simulated scenario directed towards residents in a surgical specialty, and second, the processes and strategies in which Ob/Gyn residents engage when tasked with challenging hierarchy during a clinical encounter. This is essential information needed to guide future interventions aimed towards individual behaviour change and broader organizational culture change.

## Method

### Study design

We used qualitative thematic inquiry and analysis informed by tenets of constructivist-grounded theory to answer the following socially situated research question [11]: what are the processes Ob/Gyn residents describe engaging in when faced with a simulation scenario involving an erroneous and potentially dangerous clinical decision by a medical supervisor. The research design was also informed by Tong et al.’s criteria for qualitative research [12].

### The setting

The study took place at the University of Ottawa Skills and Simulation Centre (uOSSC), a large accredited Canadian simulation centre that pursues scholarship and provides training experiences across healthcare professions and levels of training. The simulation scenario was conducted within an existing longitudinal theatre-based simulation curriculum for Ob/Gyn residents at the participating institution. This interprofessional curriculum has been operational for more than 10 years.

### Simulation scenario

This simulation scenario was conducted within an existing longitudinal simulation curriculum for Ob/Gyn residents at the participating institution. The goal of this scenario was to expose Ob/Gyn residents to a situation in which they had to respond to a supervising staff physician’s clearly erroneous and dangerous medical decision. Specifically, junior resident participants (3rd or 4th year residents) identified an abnormal foetal heart rate tracing in a labouring patient that warranted an urgent caesarean section. Prior to the scenario, the senior resident (5th year resident playing the role of the supervising physician) was instructed to take no action despite the concerning tracing. The junior resident was unaware that the 5th resident (with whom they were familiar) was a confederate or actor during this scenario, as they had been full participants during the preceding two scenarios. Thus, the junior resident was tasked with trying to advocate for an urgent caesarean section despite the supervising physician’s reluctance. This particular scenario was run as the third of three simulated scenarios during a half-day with the same team of residents. In each of the scenarios, the roles were the same (the junior resident participated as the senior resident, and the 5th year resident participated as the attending physician). The 5th year resident playing the attending physician was provided with a script to learn the night before. Some improvisation by the 5th-year resident was required to keep their responses realistic while remaining in line with the script. The 5th year residents responded similarly in all scenarios. The specific learning objectives of the clinical scenario were not expressed prior to the scenario but were incorporated into the debrief. Routine debriefing conversations followed the scenario using the Promoting Excellence and Reflective Learning in Simulation (PEARLS) debriefing framework [13].

### Recruitment and sampling

A purposive and convenience sample of eight 3rd and 4th year Ob/Gyn residents was invited to participate in a simulation scenario and a subsequent semi-structured interview. Informed consent was obtained prior to the simulation by AG and then a fellow at a Canadian simulation centre with no assessment responsibilities in relation to the participating residents.

### Research team

The research team included the following:

- GP — An Ob/Gyn, medical director of the simulation centre, and the initiator of the longitudinal Ob/Gyn simulation programme
- MDB — An anesthesiologist and active researcher in hierarchy in the healthcare setting
- AG — An Ob/Gyn and simulation fellow pursuing a postgraduate degree in health professional education
- TT — An Ob/Gyn and qualitative expert completing a doctoral degree in health professions education
- TR — A medical student at the time with an interest in simulation-based education

### Data collection

Following the simulation scenario and the routine debriefing, an experienced qualitative research assistant, with whom the participants were not familiar, conducted individual semi-structured face-to-face interviews with the eight junior resident participants lasting 30–45 min (see Table 1 for the interview guide). The questions were open ended to provide participants the opportunity to describe their experiences in depth, beyond the presented questions. The interview questions were pilot tested on members of the research team. The questions were iterated after the first two interviews. The interviews were audio recorded and transcribed verbatim. All identifying information was removed from the transcripts.

**Table 1:** Interview guide

Question number	Question
1	Can you walk me through the scenario and tell me about what you were thinking during the case? Prompts a. Was there a point when you realized something might be not quite right with the staff’s choice? (Explore) b. Was there a point that you thought to yourself, “Maybe I should say something?” (follow-up question regardless of yes or no answer). Tell me more about your train of thought at that time c. Were you initially uncomfortable saying something? Can you tell me more about why that might be? d. So, in the end, you (did/ did not) end up challenging the staff’s decision. Did you consider a scenario in your head about how you might approach dealing with the emerging situation? Can you tell me about what those scenarios were?
2	You were talking to your staff in the room/on the phone — would you have done anything different had it been the staff in fact been on the phone/in the room?
3	This was a simulation — what affect, if any, did this have on your actions/thoughts? How might you have behaved if it was a real patient?
4	In general how do you review cases with staff by phone? How do you review cases when the staff is present? Why do you think there are differences?
5	What, if anything, enables and conversely prevents you from speaking up and challenging clinical decisions about patient care made by your staff or senior colleague?
6	I’m wondering if there is anything that I haven’t asked about that you think I should know about the simulated scenario (how you felt about it? Your experience in the delivery room with colleagues? Anything you think I should know about power differential in medicine or in the delivery room in general?)

### Data analysis

Transcripts were read by the three team members who conducted the analysis (AG, TT, TR) in order to get a sense of the data as a whole. Transcripts were then analysed using an inductive approach to identifying and refining key themes [14]. Using NVivo to facilitate an organized and thorough approach to analysis, we began with manual line-by-line coding grounded in the data. Then, through constant comparative analysis within and between transcripts, we constructed more conceptual categories that captured the relationships between the initial codes. Coinvestigators AG and TT (early-career practicing obstetricians and simulation educators at the time of the analysis) independently reviewed the transcripts and constructed preliminary, line by line, codes using NVivo software. Through subsequent collaborative discussions between TT and AG, higher-order themes were constructed that captured relationships between codes. The higher-order themes were then re-examined in relation to the original narratives and to the interviews as a whole. Following this phase, co-investigator TR also independently analysed the transcripts in a similar fashion, adding a learner’s perspective to the analysis as well as adding to the credibility of the analysis. These three members of the research team then compared the results of their analyses, leading to the final list of themes when consensus was achieved. This list of themes was then checked with the long-term simulation educators and researchers (GP and MDB). An assessment of thematic saturation was also conducted [15]. In terms of thematic saturation, no new themes emerged in the last 5 interviews suggesting saturation (see Additional file 1).

### Ethics approval

Ethics approval was obtained from the Ottawa Health Science Network Research Ethics Board (no. 20150683).

## Results

### Establishing power hierarchy using simulation

All residents described the power hierarchy as a palpable challenge in dealing with the simulation scenario, whereby the supervising physician endorsed an erroneous decision. They stated it explicitly “…I think there is definite palpable hierarchy…” *(R2)*. The description of their experience demonstrates that the phenomenal mode of realism, as described by Dieckmann et al., was upheld in this scenario [16]. The scenario ‘worked’ in that the residents experienced hierarchy in line with the goal of the scenario:

I think you kind of go with, they have the clinical experience behind them and this is why they are making this decision, and I would have had a pretty similar approach to it (in a clinical situation)… Obviously I didn’t like it at the moment, but I think it’s a good scenario to reflect on how things work in a clinical situation and I think it made me reflect like how I would behave with a patient. (R1)

Furthermore, the hierarchy made semantic sense to the participants despite the fact that the attending physician was being played by a more senior resident known to the participant:

At the end, I did not challenge…I mean at the end she’s a staff so, and I’m in a training program so. (R3)

One area in which residents described a departure from realism was related to the absence of a charge nurse (in addition to the present bedside nurse) to turn to the following:

Like when I’m in labour and delivery, if this situation happened, it wouldn’t just be me, right. It would be me, the charge nurse, the nurse of the patient…In real life, I would get someone else involved. (R7)

Residents highlighted several difficulties in confronting the supervising physician. First, they described that a recognition of the power inherent in the supervisory position suppressed their own voice, “*It’s hard to…voice your concern…with a person in a position of power” (R1)*. Second, they expressed self-doubt as learners, “*maybe it’s because I’m actually wrong. I am learning, right?” (R8)*. Third, they feared negative evaluation, “*[it] will affect my ability to get a job…So I am always very conscious of being professional, polite, and not to rock the boat” (R2)*. Fourth, they raised concerns about patients’ trust in staff, “*You don’t want to discredit your staff too… because then that would take the confidence away from the patient” (R7)*.

### Communication strategies and coping mechanisms within the context of hierarchy

**Fig. 1:**
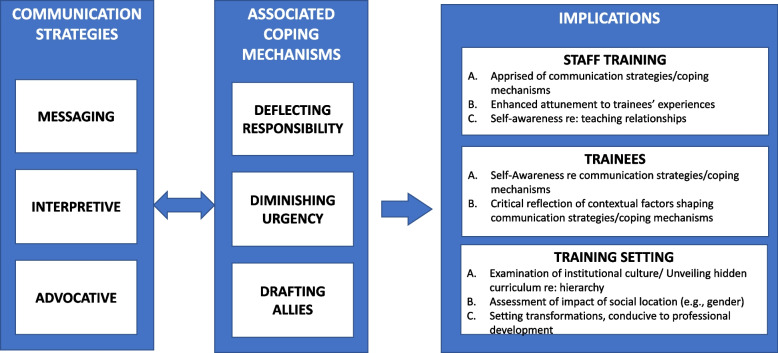
Residents’ communication strategies and associated coping mechanisms in response to challenging hierarchy and potential study implications

Through iterative stages of analysis, three communication strategies were identified across the interviews in relation to residents’ challenges in confronting an erroneous supervising physician. These included the following: (a) messaging strategies, (b) interpretive strategies, and (c) advocative strategies (see Fig. 1). The analysis further revealed a number of coping mechanisms residents employed to deal with the stress engendered by this situation, namely deflecting responsibility, diminishing urgency, and drafting allies (Fig. 1). Below, we expand on the predominant communication strategies and delineate related coping mechanisms by providing salient quotations from the interviews (identified by participant code). Additional examples are provided in Additional file 1.

### Messaging strategies

When utilizing messaging strategies within the healthcare team, residents focused on conveying the existing clinical information to the staff physician without the intention of influencing the staff’s decision making.

… okay, I feel like I've communicated the situation. I feel like I've described the tracing... I guess I trusted that, based on what she knew, the decision she was… what she was telling me was grounded in something. (R6)

As a coping mechanism, residents who primarily relied on messaging strategies often deflected responsibility for the patient’s safety with deference to the staff physician:

… ultimately, they’re the most responsible physician so if they’re deciding to wait, then I mean they’re taking that responsibility on, right? It’s my job to maybe inform them of what’s happening. So as long as I do that, at the end of the day it is their decision (R4).

Messaging strategies were not intended to necessarily influence the final treatment decision, and thus, having conveyed the correct information, some residents felt that their duty was fulfilled at this point, leaving the staff physician to interpret and act on the information.

### Interpretive strategies

When utilizing interpretive strategies to challenge the patient safety threat, residents took a more persuasive approach. In such cases, residents sought to lead their staff in the direction of what they believed to be the correct clinical decision. Interpretive strategies, unlike messaging strategies, were more deliberate and emphasized a different interpretation of the clinical facts from the perceived interpretation of the staff physician:

So how do I communicate that I’m concerned without saying I don’t trust your clinical judgment…how do I convince them that this is concerning so that they come up with the decision on their own that this is concerning. Right? (R1)

Because of their relatively subordinate position within the hierarchy, residents were conscious of their language when delivering an interpretive message to the staff physician:

…be careful in the sense of not using any words too explicit, like ‘this baby might die’… trying to increase the urgency without saying any direct statements that may amplify the situation (R2).

When interpretive strategies failed to convince the staff to pursue their preferred course of action, residents employed coping mechanisms to mitigate the resulting tension. Residents minimized their own sense of urgency regarding the case:

I think that, you know, if we had acted at that very moment that I said that I want to proceed with a C-section and within thirty minutes I think chances are that outcomes would have been sort of exactly the same. (R2)

When utilizing an interpretive approach, residents also described deflection of responsibility as a coping mechanism:

If I can’t convince them to do it the way I think to do it, well, my kind of position as learner is to then back out of it and say well, now it’s yours. And then they assume the entire responsibility and take away my responsibility at that point (R8)

By diminishing urgency and deflecting responsibility, residents were able to rationalize their compliance with a scenario that clearly threatened patient safety despite their best efforts to sway the staff physician’s interpretation of the case.

### Advocative strategies

Advocative strategies were the most active of all the tactics described by residents when faced with managing a dangerous clinical decision made an attending physician. Residents were motivated to use these strategies out of a sense of responsibility to the safety of the patient despite opposition by the supervising physician. When employing advocative strategies, residents utilized language that conveyed their ownership of patient care and outcomes:

…I’m the one who’s doing everything… like I feel like I’m with the patient the whole time, right. I see them in triage. I’m with them in labour and delivery and the staff is there too, but they’re there as a manager…I just feel so responsible for their wellbeing that I feel like I can’t let this happen, someone needs to speak up. (R7)

Such a sense of ownership led residents to take more active steps towards proceeding with a caesarean section without the support of the supervising physician:

You would just have to be like okay. Well we’re moving her back now. Whether you call in your second or you do it yourself, I don’t care… I couldn’t watch a baby die. (R5)

However, residents also acknowledged the discomfort inherent in occupying this role*,* admitting that, “I was uncomfortable with all the language that I had to use” (R5). It seemed that, when using the advocative approach, the source of discomfort was about not only threatening the hierarchy but also concern that taking action might fracture patients’ confidence in the system:

…you don’t want the patient to think that, oh, this team is not even on the same page (R7).

Residents resorted to drafting allies as both an approach to advocacy and a mechanism of coping with the inherent challenge of confronting the staff physician directly. Residents described seeking out second opinions from the nursing team or other colleagues to leverage their stance:

I would have gotten the charge nurse involved, right, and they’re respected a lot by the staff and I feel like maybe their opinion, along with mine, would drive the message across (R7).

I suggested that maybe first we can get a consult from high-risk team (R3)

Residents also described enlisting nurses as a coping mechanism to combat their own self-doubt:

The main thing is the confidence in your own position…if you have the support from the nurses…well then you go well, I’m not the only person on my island who’s thinking like this. (R8)

Therefore, when using advocative strategies, residents did not shy away from using escalating language to advance the dialogue with their supervising physician. They persisted in seeking out a solution that ensured a safe patient outcome, regardless of whether they could convince the supervising physician to take appropriate action. They drafted allies to both aid their advocacy and cope with their sense of doubt.

## Discussion

Towards the broader goal of contributing to existing research about challenges of confronting hierarchy among surgical trainees in relation to patient care, the study aimed to examine both the utility of hierarchy-focused simulation scenarios in addressing such challenges, as well as the processes and strategies in which trainees engage when tasked with challenging hierarchy to optimize patient care and avoid adverse outcomes. Related to the first aim, study results show that we were successful in creating a sense of hierarchy in a simulated setting. This is in line with Calhoun and colleagues who found that they were able to use simulation to address hierarchy-related medical errors among anaesthesia residents [17]. In our study, the semantic and phenomenal modes of realism were upheld despite utilizing senior resident peers, known to the participants, as the confederates playing the role of the supervising physician. One aspect of the scenario that detracted from the realism was the absence of a ‘charge nurse’ that residents would have liked to lean on for support during this interaction. The study therefore supports existing work in suggesting that simulation can be used to further explore and teach around this challenging topic in a safe educational setting.

Related to the second aim, the study highlights a spectrum of communication strategies employed by Ob/Gyn residents when faced with a threat to patient safety in the context of simulated hierarchy. Residents adopted three distinct communication strategies, namely messaging, interpretive, and advocative. While messaging strategies involved a mere dispassionate reporting of existing clinical information, interpretive strategies reflected a deliberate construction of the clinical facts aimed at swaying the staff physician’s clinical decision. Advocative strategies involved a guarded readiness to confront the erring staff physician while employing language that conveyed ownership of patient care and outcome.

These findings have a number of training-related implications in relation to teaching staff, trainees, and the training setting as a whole (see Fig. 1). First, in relation to training staff, clinical teachers who are apprised of the emergent communication strategies may be more equipped to recognize the layered language that can accompany clinical facts when residents work to convey information across a hierarchy. For example, teaching staff can become attuned to the degree of safety they provide in their learning environment by assessing their trainees’ communication strategies. The use of advocative strategies may suggest an environment of safety. On the other hand, being aware of receiving communications from residents that rely exclusively on messaging strategies could invite clinical teachers to examine the nature of the dynamics of their teaching relationships. Furthermore, within this context, receiving a rare advocative communication may imply that the trainee has sufficient concerns regarding a clinical decision to take on significant interpersonal risk by speaking up. Being apprised of trainees’ use of communication strategies can therefore enhance self-awareness among faculty about their teaching relationships.

Second, residents themselves may find it useful as well to become aware during simulation debriefings of the relational patterns that unfolded within the hierarchical structure. Not only can such greater awareness mobilize learning but also it invites residents to examine contextual factors, such as age, gender, ethnocultural heritage, and other factors that may shape their processes of learning. To date, curricular initiatives have focused on providing residents with particular communication tools to build up their skill set when speaking across hierarchy [18, 19]. Some have shown effect, while others have not [20]. The spectrum of communication strategies found in this study implies that such tools and interventions might be most effectively incorporated once residents possess insight into their own experiences when tasked with speaking up across hierarchy. A simulated case such as described in this study can provide an opportunity for such debriefing conversations informed by the emergent communication strategies.

Third, beyond informing specific teaching faculty and trainees, the study can provide tools for examining the hidden curriculum regarding power and hierarchy at the training setting. This includes the examination of the workplace environment as a whole, including the complex interplay of contextual factors. For example, lower local safety culture scores correlate with lower self-reported efficacy of speaking up [21]. Furthermore, residents’ responses to hierarchy are inevitably shaped by several complicating contextual factors. In one simulation study, a female attending physician was more likely to be challenged by a respiratory therapist, and that challenge was more likely to be assertive and effective suggesting significant gender influences [22]. In a study of two academic birth centres, Lyndon found that interpersonal relationships between providers could impact their willingness to voice concerns, thereby influencing quality of care and patient safety [23]. Beyond providing tools to examine hierarchy-related processes, the study also suggests the importance of further study among multidisciplinary interprofessional teams and calls for curricular interventions that target culture, span levels of training, and work towards adjusting the perpetuation of the negative components of hierarchy while preserving the positive aspects.

Woven into the range of communication strategies, we identified coping mechanisms that helped the residents mitigate tensions related to the challenge of confronting hierarchy. The identified coping mechanisms included the following: deflecting responsibility, diminishing urgency, and drafting allies. Both diminishing urgency and deflecting responsibility seemed to represent internal mechanisms that enabled residents to relieve tension related to their inaction despite the risk of an adverse outcome while utilizing messaging or interpretive communication strategies. Drafting allies represented a more active coping strategy invoked by residents using advocative strategies in order to reduce associated experiences of fear and isolation. Our findings are in line with Bould et al. who described the coping strategies of conflict avoidance and diffusion of responsibility [8].

The coping mechanisms found in the present study, particularly diminishing urgency and deflecting responsibility, raise concerns as they directly contradict the goals of training [24]. As residents progress through training, their ability to recognize urgency and take ownership of cases should grow. Finding that hierarchy can have the unintended effect of inhibiting this process is important. The drafting of allies is perhaps more nuanced in that it is a collaborative strategy; however, leaning on colleagues for validation at a time of medical emergency may present challenges as well. Like communication strategies, insight into these coping mechanisms can contribute to training (Fig. 1) by deepening coaching and debriefing conversations around crisis resource management and professional identity formation. Furthermore, increased awareness of these coping mechanisms can invite dialogues about the institutional context, such that constructive changes to the greater setting can be considered and implemented.

This study was conducted with a qualitative methodology that provides rich, in-depth insights but limits generalisability. The main limitation of the study relates to the number of participants. Though we reached thematic saturation, in that no new themes emerged after the interview with the third participant, we acknowledge that the small number of participants limited the study of contextual factors and their potential shaping of response patterns. Moreover, thematic saturation, as a positivist construct, has been challenged, and future work on this subject might employ other frameworks such as meaning or theoretical saturation going forward [25, 26]. It is also important to acknowledge that the interviews were carried in the weeks following the simulation. A routine debriefing conversation was carried out immediately after the simulation. The debriefing conversations were learner centred and not prescriptive. Although the debriefing could have impacted some aspects of the responses, the responses speak to a clear distinction made by the participants between their behaviour and experiences during the simulation and their reflections during the debriefing. We suggest that this study be implemented in a number of training sites, allowing comparison of emergent findings across a number of settings as well as providing the opportunity to have a larger number of participants so that the impact of contextual factors, such as gender, age, or ethnicity, can be studied. Further study should also examine the workplace environment and the role of faculty physicians in supporting a workplace in which it is safe to speak up.

## Conclusion

This study suggests that simulation can be successfully utilized to explore the experiences of surgical residents as they navigate hierarchy in the face of a threat to patient safety. Simulation can therefore be used both for the continued study of the topic and for teaching about this challenging topic in a safe educational setting. In addition, the qualitative study brings to the fore a spectrum of communication strategies employed by residents as they experience challenges in confronting the power hierarchy within the context of compromised patient care. Furthermore, the study explicates associated coping mechanisms aimed at mitigating anxiety about confronting the power hierarchy within a surgical specialty. These communication strategies and coping mechanisms can serve as tools for both teaching faculty and residents by enhancing self-awareness, providing a way to assess safety of the teaching relationship, and inviting an examination of related contextual factors. Furthermore, the findings suggest the value of going beyond skill-based interventions of confronting hierarchy and explicating the hidden curricula in relation to challenging the power hierarchy in the setting as a whole. Such explorations can deepen coaching conversations towards providing an educational culture which nurtures professional development.

## Supplementary Information

**Additional file 1.** Themes and sample illustrative quotations of the communication strategies and coping mechanism categories.

## Data Availability

The datasets used and/or analysed during the current study are available from the corresponding author on reasonable request.

## Abbreviations

PEARLS: Promoting excellence and reflective learning in simulation
uOSSC: University of Ottawa Skills and Simulation Centre
Ob/Gyn: Obstetrics and gynecology
