# Supplementary material for: Facing hierarchy: a qualitative study of residents’ experiences in an obstetrical simulation scenario
Source: Adv Simul (Lond). 2022 Oct 23;7:34. doi: 10.1186/s41077-022-00232-1 (PMC9590210; doi:10.1186/s41077-022-00232-1)
Supplement: Supplementary file 1 — Additional file 1. Themes and sample illustrative quotations of the communication strategies and coping mechanism categories. [file 41077_2022_232_MOESM1_ESM.docx]

Additional file 1

Themes and sample illustrative quotations of the communication strategies and coping mechanisms categories

| Categories | Themes | Sample citations/narratives (sample) |
| --- | --- | --- |
| Communication Strategies | Messaging | At the end of the day you document facts and at the end of the day the staff is the most responsible physician. (**R3**)  It’s my job to maybe inform them of what’s happening. So as long as I do that, at the end of the day it is their decision. I guess this is the way I see it. (**R4)**  So you want to get through the bullet points quickly. So who they are. What’s the issue. What you want do about it in that order. Concise and to the point… just summarize and go through it (**R8)** |
|  | Interpretive | So if I’m reviewing a patient with the staff, I will cut out all the stuff that doesn’t add up and just say, say somebody is coming in with an infection, I would say, “She has a fever, she has a white count and she looks ill.” So I kind of biasedly present all the bad things and try to avoid the good things just so that they have a clear picture that I’m very worried. (**R2**)  You start to become more liberal with your language and your persuasiveness, …, by saying things like, ‘the tracing’s abnormal, this baby could be in danger.” (**R5**)  I would, you know, ask questions, kind of trying to point them towards… to think more towards me. Like pointed, kind of leading questions to kind of see can I get them to kind of start maybe thinking about it differently by asking the right questions. (**R8)** |
|  | Advocative | “I’m very concerned, what do you think we should do next? I’m not comfortable with this”, and they were still sort of trying to say, like, “no it’s fine, I’m okay, let’s just keep going” and then I was sort of saying, like, “no I’m not comfortable, I think we should do a Cesarean section on this woman. I don’t think that the fetal heart rate, I believe that the fetal heart rate is abnormal.” (**R1**)  “Saying things like, ‘the tracing’s abnormal. I think we should move forward with a C-section’… I did get to the point where I said maybe we could have a second opinion” (**R5**)  I want to be respectful in workplace and respect my staff and my colleagues, like patient safety has to be first, right. I can’t let anything else trump patient safety. So I feel like if my… if at the bottom of my heart what I’m doing is for the safety of the patient, then no matter what happens, like if they think I’m rude or whatever, I think it’ll be okay in the end, because it’s for the right reason, right. (**R7**) |
| Coping mechanisms | Deflecting responsibility | We sort of rely on the staff as the final decision…  -They might say yes or no, but in the end it was their decision, it wasn’t my decision. (**R1**)  So it’s not that you don’t care about the patient, but you can only suggest things and the staff is the one who makes the call (**R3**)  I had told the patient, “I'm going to talk to my staff… my supervisor and see what they think.” And I think when I came back, I was like, “Well, I talked to Dr. so and so and they’re… you know, they're aware of the situation and they're okay with waiting” (**R6)** |
|  | Diminishing urgency | When I called them the first time, even I was like, oh, maybe we could wait a little bit longer because the heart rate had just started to be abnormal **(R1)**  I was kind of willing to accept the fact that the staff wasn’t gonna proceed with the C-section because I was hoping that in due time, within a certain amount of period, they would sort of change their mind. (**R2**)  So then initially my thought was not to override the staff’s opinion. So my thought was ‘Okay, If they want to manage this expectantly then we’re going to watch it closely and we’re going to see what happens’, which I think is sometimes reasonable, especially in the birthing unit when, you know, crappy tracings do recover. (**R4)** |
|  | Drafting allies | And I think I suggested that maybe first we can get a consult from high risk team. We can get… get another opinion. (**R1**)  I would have gotten my senior to help me out, and you know, as a second voice, as someone who, you know, maybe has a more long-term relationship with the staff and they know each other better (R5)  I think that sometimes what is helpful, what has been helpful for me is also to really like also get the opinion of the nurses too. And say like, “Listen, this is the situation, I'm not super comfortable with this. Dr. so and so doesn’t seem to be too worried. What do you guys think?” (**R6**) |

Appendix A outlines the identified categories and themes and a sample of 3 narratives for each theme that were not included in the manuscript. Note: all themes within the two categories emerged by the third interview.
